# Monthly Summary

**Published:** 1883-03

**Authors:** 


### MONTHLY SUMMARY.

*The Use of Tobacco by Children.*—Considerable attention is being paid both in the medical and lay press to the subject of tobacco-using among young lads. A well-known female correspondent has made the statement that seventy-five per cent. of school-boys over twelve or thirteen years old smoke cigarrettes probably without the knowledge of their parents, but not unknown to the teachers. She says further that the principal of one of the largest private schools in the country assured her of its pronounced evil effects upon his boys, but that he was so convinced of the firm hold the habit had gained upon them that he considered it as time thrown away to remonstrate or interfere with it. The principal of a grammar school in a neighboring city found on investigation that of the boys under his charge, in age from eight to fifteen, four fifths confessed to using tobacco in some form. The deleterious effects of the drug in arresting the development of children are sufficiently known, but perhaps their importance is hardly realized. A correspondent of the *British Medical Journal* describes a case in which arrest of growth in the organs of generation seemed to be due to this, cause. He was twenty five years of age, and five feet nine inches and a half in height. His penis was small and the prepuce rather long, but not in a condition of phimosis. The testicles were remarkably small, neither being larger than a french bean or, perhaps what more nearly expresses their size and shape, no larger than the testes of a rabbit. The scrotum was not relaxed nor was there any varicocele. There was short dark hair on the pubes. The voice was somewhat high pitched, yet not like that of a woman or eunuch. Though twenty-five, he had not a trace of a beard, whisker, or mustache; nor was there any hair on the chest or around the nipples. The breasts were flat, yet the contour of the lower extremities and lower part of the trunk resembled that of a woman more than that of a man.

In observing this man one could but be struck with the evidence of development on a manly type up to a certain period, and then of a cessation of further virility. His manly height and muscular development ill accorded with the entire absence of beard and weakness of expression. In these respects he differed from the short, rounded, plump eunuchs produced by robbing children early of their testicles. In searching for a cause of this arrest of development, we ascertained that he had not suffered from mumps, nor from any inflammation of the testes. There appeared, indeed to have been no disease which could have checked the growth of these organs. He, however, stated that he was an inveterate tobacco-chewer, preferring a good "quid" to victuals; and that he commenced this habit at the early age of nine, by stealing from his father's pouch what he could not afford to buy. His mouth, at the time of examination, was occupied by a "plug," and there was further evidence of the habit in the staining of the teeth where the dentine was exposed. In the absence of any other cause to which this condition of generative organs could be referred, the writer was inclined to attribute their arrest of development to the poisonous effects of excessive chewing of tobacco.—*Boston Medical and Surgical Journal.*

---

*An Idea on Amalgams.*—One great objection to amalgams is that they shrink and turn black, consequently stain, disfigure and destroy the organs that you are trying to beautify and preserve. That heat expands metallic substances is a fact well known to the most casual observer, the expansion being a rupture, a breaking apart of the crystals of which it is composed, —this being nothing more or less than the annealing process of all metals; but if this process goes into a *vacuum*, and by annealing and re-annealing, and subsequent melting, you have then a product that does not undergo such changes.

Annealing of metals in *free air* tends to soften them, for the reason that has been given, *i. e.*, cracking; whereas this new method of annealing produces an opposite effect, making metals harder, more ductile, and what is of greater importance, their density is entirely changed. Then by amalgamating with pure mercury (just so much and no more) you should have but little or no shrinkage, and no oxydation whatever. Now when this amalgamation takes place, more or less heat is evolved, but i this heat is allowed to depart before inserting the material in the cavity, it will *not shrink*, because crystallization at *low temperatures* is not a process of contraction. This is easily proven in observing the crystallization of water, "freezing," and the consequent bulging or bursting of pipes or vessels in which it is contained. Hence, the laws which govern all substances in passing from a fluid, or a semi-fluid, to a solid state, and the laws that control these substances during the process of crystallization, should be thoroughly understood by all operators. So, upon the proper choice of materials, and intelligent manipulation of them, depends the success or failure of a *plastic* for filling and preserving teeth. And without this knowledge, and the action and numerous changes these substances undergo during the varions processes of manipulation, your labor and time is lost.—*Ohio State Journal of Dental Science.*

---

*Carbolic Acid Poisoning.*—Dr. Ingessii in *Bulletin General de Therapeutique*, has arrived at the following conclusions respecting this: First, the symptoms of poisoning by external application of carbolic acid are the same as those which arise from the absorption of the poison by the stomach from the gastric mucous membrane. Second, poisoning occurs certainly where the acid has been applied to the skin or injected into a serous, mucous, or abscess cavity. From the exposed surface of a wound the absorption is very slight, and the toxic effects trifling. The mucous membrane of the respiratory passages may serve as the place of introduction of the poison. Third, the effects may assume a very acute form, a less acute form or a chronic form. Fourth, there exist certain idiosyncrasies; women and children are especially liable to carbolic acid poisoning. Fifth, the toxic dose is variable. In persons predisposed, on grain of carbolic acid may be sufficient to poison. Sixth, carbolic acid as an application to contused wounds should be used with caution, and in some cases should even be substituted by a less dangerous agent. Seventh, the treatment of severe carbolic acid poisoning should consist in artificial respiration and diffusible stimulants, especially the hypodermic injection of ether. In other cases the removal of the cause, through the discontinuance of the remedy, will suffice to remove the symptoms.—*Druggists' Circular.*

---

*A New Hypnotic and Depresso-Motor.*—Herr Schiffer, of Berlin, has recently been studying the action of guachamca, a Venezuelan plant. It acts very much like curare, in allaying spasm, and in large doses causing a general paresis. The juice taken from the plant in the rainy season makes the most powerful preparation. If ten milligrammes of the extract are given to a frog, an interval of fifteen to eighteen minutes passes before any effect is noticed. After this period, however, the action is rapid. The animal becomes stupid, allows the head to fall, permits itself to be laid on its back, and does not draw back the leg if extended, etc. So far its action is exactly similar to that of curare; but now comes the difference. The respiration continues, the circulation and cardiac activity are undisturbed. Its effect was tried on a young man in Frerich's clinic who was suffering from cramps. Ten milligrames of the extract were injected, but for three-quarters of an hour no effect was observed The patient then, in broad daylight, suddenly fell into a rather deep sleep, which lasted for nearly three hours; respiration and circulation were undisturbed. Schiffer believes that in guachamaca we have an agent capable of combating disorder of the motory apparatus, as well as a useful hypnotic.—*Medical Record.*

---

*Influence of Electric Light on Health.*—The influence of the electric light on health was lately discussed at a meeting of the Hygienic Society of Hamburg, and Dr. Kruss, gave his views on the subject at some length. He referred to the influence of the electric light on the human eyesight, and expressed his opinion that it produces no evil effects, the light having a violet tinge under most circumstances. The electric light being free from the disadvantages incidental to the combustion of gas, in the comsumption of oxygen and the production of carbonic acid, he considered its development as being a hygienic measure of importance.—*Med. and Surg. Reporter.*

